# Application of the Highway Safety Manual-2010 to expect average crash frequency at full cloverleaf interchange

**Published:** 2019-08

**Authors:** Kamran Rahimov, Sadegh Samadi, Mohammadreza Neghabi

**Affiliations:** ^*a*^Tehran Payam Noor University, Tehran, Iran.; ^*b*^Tehran–Azad University, Tehran, Iran.

## Abstract

**Background:**

In this study, suggested pattern of HSM-2010 has been developed to expect crashes on Azadegan Interchange in the Tabriz, Iran. IHSDM – HSM software was used to predict and expect the average crash frequency, crash severity, and crash type distributions.

**Methods:**

The site that evaluated in this study was a full cloverleaf interchange in an urban area. It is a service Interchange at the junction between a freeway and a minor arterial cross Street. When using the Predictive, the freeways and ramps within the defined project limits were divided into individual sites. A site is a homogeneous freeway segment and homogenous ramp segments. The focus of the discussion in this study is on the sites located in the vicinity of an interchange due to the complexity of interchange roadways.

**Results:**

The results of this study showed that the calibration factors obtained for this interchange were 3.68 for FI crashes and 1.35 for property damage only. It was also observed that using accident statistics occurred at the Azadegan interchange by 2016 and 2017 as well as the geometric characteristics of the interchange, the total expected average crash frequency by 2018 is 13.70, of which 7.8 cases were fi crashes, and 5.9 cases are property damages only. It was also observed that in the freeway segment, the severity of the expected fatal injuries, incapacitating injuries and non-incapacitating injuries, decreased by approximately 95, 86 and 4% respectively, and the expected possible injuries and the property damage only ( no injury ) increased by 156% and 239%, respectively. Also, in the entrance and exit ramps of the interchange, the expected severity of the fatal injuries, incapacitating injuries and non-incapacitating injuries, possible injuries and the property damage only ( no injury ), decreased by approximately 98, 94, 53, 24 and 1% respectively.

**Conclusions:**

Use of highway safety manual to assess the interchanges, provide useful information to predict and expect the average crash frequency, crash severity, and crash type distributions.

**Keywords:**

Full Cloverleaf Interchange, IHSDM – HSM, Expected Crash Severity, Expected

